# Pharmacological Potential of Selenoproteins in the Regulation of Oxidative Stress in Liver Diseases

**DOI:** 10.3390/pharmaceutics18091167

**Published:** 2026-09-16

**Authors:** Elena G. Varlamova

**Affiliations:** Institute of Cell Biophysics of the Russian Academy of Sciences, Federal Research Center “Pushchino Scientific Center for Biological Research of the Russian Academy of Sciences”, St. Institutskaya 3, 142290 Pushchino, Russia; 1928lv@mail.ru

**Keywords:** liver pathologies, targeted pharmacotherapy, reactive oxygen species, oxidative stress, selenoproteins, antioxidants

## Abstract

**Background:** Pathological activation of oxidative metabolism is a universal catalyst for liver parenchyma destruction. While physiological pools of reactive oxygen and nitrogen species (ROS/RNS) regulate hepatic regeneration and cellular respiration, decompensation of endogenous antioxidant systems induces cascading damage across hepatocytes, sinusoidal endothelial cells, Kupffer macrophages, and hepatic stellate cells. Current therapies against liver fibrosis, cirrhosis, and hepatocellular carcinoma remain suboptimal, necessitating the identification of novel, druggable molecular targets. **Focus:** This review synthesizes current evidence on ROS-driven necroinflammatory and degenerative cascades and evaluates the multi-level protective potential of the selenoprotein family. **Evidence:** Selenoproteins function as a highly heterogeneous network where specific members are vital for cell survival (e.g., GPX4 in preventing ferroptosis), while others exhibit stage-dependent expression throughout progression from inflammation to malignancy. **Conclusions:** In addition to directly scavenging free radicals, selenoproteins modulate endoplasmic reticulum stress, metabolic remodeling, and immune responses. A more complete understanding of the important role of selenoproteins in regulating liver pathological processes, particularly their antioxidant function, allows them to be considered as promising potential targets for the further development of pharmacotherapeutic strategies for the treatment of chronic liver diseases.

## 1. Introduction

The oxidative cascade is a fundamental trigger for liver tissue destruction, leading to organ dysfunction and subsequent apoptosis of its cellular structures. Reactive oxygen species (ROS) and reactive nitrogen species (RNS) are produced in normal hepatocytes and are critical for normal physiological processes, including oxidative respiration, growth, regeneration, and apoptosis. When the level of oxidative products exceeds the capacity of normal antioxidant systems, oxidative stress occurs, leading to damage to liver cells, including hepatocytes, Kupffer cells, stellate cells, and endothelial cells, through the induction of inflammation, ischemia, fibrosis, necrosis, apoptosis, or malignant transformation through damage to lipids, proteins, and/or DNA [1,2,3,4]. Disruptions in the functioning of endogenous defense systems significantly accelerate the development of degenerative processes in the hepatobiliary system. Moreover, pathologically high concentrations of free radicals are recorded in all forms of protracted liver damage, regardless of their etiology. To neutralize them promptly, the liver parenchyma deploys a complex set of regulatory enzymes, including various pools of superoxide dismutases, peroxy- and thioredoxins, as well as selenium-dependent glutathione peroxidases and thioredoxin reductases [5,6,7,8,9,10,11,12,13,14,15,16].

Cellular pools of selenium-containing proteins play a critical role in regulating redox balance, directly controlling the level of destructive radicals. It is now well known that antioxidant function is one of the key functions of mammalian selenoproteins, but it is far from the only one [17,18,19,20,21,22,23,24,25,26,27,28,29,30,31,32,33,34]. Furthermore, it has been repeatedly shown that mRNA expression and selenoprotein activity may differ depending on the stage of disease in a particular organ, including the liver [35,36,37]. A comprehensive analysis of experimental data in rodents demonstrates that the metabolic significance of selenoproteins varies significantly across individual cells, making it unacceptable to consider this protein family as a single functional monolith [38,39,40,41]. Deficiencies of some selenoproteins completely block liver function, while the absence of others is compensated for by alternative pathways.

The pharmacological potential of selenoproteins opens new prospects for targeted therapy of various liver pathologies, as these proteins are capable of directly blocking necroinflammatory cascades. For example, in fatty liver disease and steatohepatitis, targeted stimulation of glutathione peroxidase enzymes minimizes lipotoxicity and inhibits oxidative membrane degradation. In cases of alcohol-induced liver damage, where the mitochondrial matrix is the primary target, the use of organoselenium mimetics restores the depleted thioredoxin pool, protecting the parenchyma from toxic ethanol metabolites. Selenoproteins play a special role in preventive hepatic oncology and the fight against fibrogenesis. Stabilization of the redox status with selenoproteins prevents the phenotypic transformation of stellate cells, significantly slowing the excessive deposition of collagen fibers and preventing cirrhotic remodeling. In chronic viral infections, maintaining optimal selenium concentrations in tissues modulates the immune response and reduces the rate of pathogen replication. In the early stages of carcinogenesis, certain fractions of these proteins act as natural tumor suppressors, protecting nucleic acids from the mutagenic effects of free radicals and reducing the risk of malignancy.

This review examines liver pathologies, beginning with steatosis (fatty hepatosis), which is the initial stage of Metabolic Dysfunction-Associated Fatty Liver Disease (MAFLD), in which excess triglycerides begin to accumulate in liver cells. Inflammation is absent, liver function is preserved, and the process is reversible. If fat in hepatocytes becomes excessive, steatohepatitis (MASH, metabolic dysfunction-associated steatohepatitis) develops, a type of hepatitis caused by the toxic effects of fatty deposits. Also discussed is Alcohol-Associated Liver Disease (ALD), in which the trigger is chronic alcohol consumption. This can subsequently lead to alcoholic steatohepatitis. Hepatitis can also be caused by other external factors (viruses, toxins, etc.). With timely treatment, various types of hepatitis are reversible. Chronic inflammation, characteristic of hepatitis, causes the liver to constantly heal damage, causing the organ to develop hard connective tissue, which is partially reversible in the early stages. The final stage of fibrosis is cirrhosis, in which scar tissue completely alters the liver’s structure, blocking blood flow, leading to loss of liver function, and is irreversible. The review will also discuss liver cancer (hepatocellular carcinoma), which has various etiologies but can develop due to chronic cellular damage, inflammation, and mutations in hepatocytes.

This review describes the current understanding of the role of ROS in the progression of various liver pathologies, as well as the functions of selenoproteins that mitigate these processes. Particular attention is paid to the antioxidant function of these proteins. The relevance of selenoprotein research in modern hepatology is driven by the need to discover fundamentally new therapeutic molecules, as standard therapeutic approaches are often unable to halt the progression of fibrosis, cirrhosis, HCC, and other pathologies. The antioxidant role of selenoproteins in this context lies in the formation of a multi-layered protective barrier capable of intercepting and deactivating destructive radicals at various stages of their generation. The review clearly demonstrates that the expression patterns of selenoproteins vary significantly depending on the liver disease. In addition to their antioxidant function, they perform a number of other important functions aimed at mitigating or exacerbating pathological processes.

The approach for writing this review was to select the relevant topic of “Various liver pathologies” with a focus on one of the key causes: excessive or impaired ROS generation, as well as the role of selenoproteins, whose important antioxidant function is present in these processes. Since no similar review has been published in the past two years, and new experimental data are constantly emerging, this review focuses on analyzing this problem. Initially, a vast literature review covering the past five years was conducted, and a list of key terms was compiled: liver pathologies and ROS, liver pathologies and selenoproteins, selenoproteins as antioxidants, redox status impairment in liver pathologies, etc. Reputable platforms were used for literature analysis: PubMed/MEDLINE, Scopus, Web of Science, Google Scholar, CyberLeninka, and eLibrary.

## 2. Non-Alcoholic Fatty Liver Disease (NAFLD) or Metabolic Fatty Liver Disease (MAFLD)

Metabolic fatty liver disease (MAFLD), a new nomenclature introduced in 2020 to designate fatty liver disease instead of non-alcoholic fatty liver disease (NAFLD), remains one of the leading causes of end-stage liver disease. This disease represents a spectrum of liver disorders defined by macrovesicular steatosis in ≥5% of hepatocytes in the absence of secondary causes such as excessive alcohol consumption [42,43,44,45,46,47]. MASLD is becoming increasingly common, particularly in the Middle East and Western countries, as the prevalence of obesity increases. It is the most common form of liver disease worldwide. MASLD ranges in severity from hepatic steatosis—sometimes called diffuse hepatic steatosis, or previously fatty liver, or simply fatty liver—to a more severe form of the disease called metabolic dysfunction-associated steatohepatitis (MASH), formerly known as nonalcoholic steatohepatitis (NASH). Recently, mounting evidence suggests that intestinal dysbiosis plays a significant role in MAFLD through the regulation of inflammation, insulin resistance, bile acid, and choline metabolism [48,49,50,51,52]. Patients with MAFLD typically have small intestinal bacterial overgrowth (SIBO), which can disrupt intestinal tight junctions, increase intestinal permeability, and stimulate an inflammatory response. Intestinal dysbiosis is responsible for increased lipopolysaccharide (LPS) secretion and lipopolysaccharide-mediated inflammation in the development of MAFLD.

### 2.1. The Role of ROS in the Development of MAFLD

There are a number of sources that generate excessive ROS formation, contributing to the development of MAFLD. It is well known that mitochondria play a significant role in the oxidative stress observed in MAFLD, with one of the most well-known sources being mitochondrial oxidative phosphorylation, which produces superoxide anions as byproducts [53,54,55]. Excess mitochondrial Ca^2+^ can also significantly contribute to the formation of mtROS, which ultimately contributes to mtDNA damage, further accelerating the development of MAFLD.

The endoplasmic reticulum (ER) is considered another source of ROS, where electron transfer by cytochromes P450 in the CYP cycle is impaired, leading to the activation of oxygen molecules before substrate binding [56,57,58,59]. As a result, oxygen is reduced not to water, but to reactive ROS, such as superoxide anions and hydrogen peroxide, and in the presence of divalent iron ions, peroxide can be converted into a more toxic hydroxyl radical. Prolonged ER stress can also cause oxidative stress in the liver [22,60]. In addition, the catalytic process of Ero1 oxidoreductase and NADPH oxidoreductase occurs in the ER, which is also a source of ROS. It was shown that high expression of Ero1 promoted the accumulation of hydrogen peroxide, caused ER peroxidation, which led to the aggravation of MAFLD [61]. ROS in the ER can also be formed as a result of the oxidation of thiol groups during oxidative folding of proteins. In this case, disulfide bonds are formed in proteins, and hydrogen peroxide is formed as a by-product [62]. During ER stress, the expression and activity of the NADPH oxidase (NOX) family of proteins in hepatocytes, hepatic stellate cells, and Kupffer cells are also increased, which promotes the oxidation of NADPH, which is accompanied by the generation of superoxide and hydrogen peroxide [63,64].

Furthermore, hydrogen peroxide can also be formed during peroxisomal β-oxidation, subsequently converting to a hydroxyl radical. Peroxisomes are important organelles where lipid metabolism occurs, particularly the oxidation of very long-chain fatty acids [65,66,67,68]. Acyl-CoA oxidase 1 (ACOX1) is an important step in peroxisomal β-oxidation, catalyzing the desaturation of acyl-CoA to 2-trans-enoyl-CoA, leading to the formation of hydrogen peroxide [69,70].

The level of prooxidants and ROS also increases with inflammation and xanthine oxidase activation, high levels of which are also characteristic of MAFLD [71,72,73,74].

**Figure 1:**
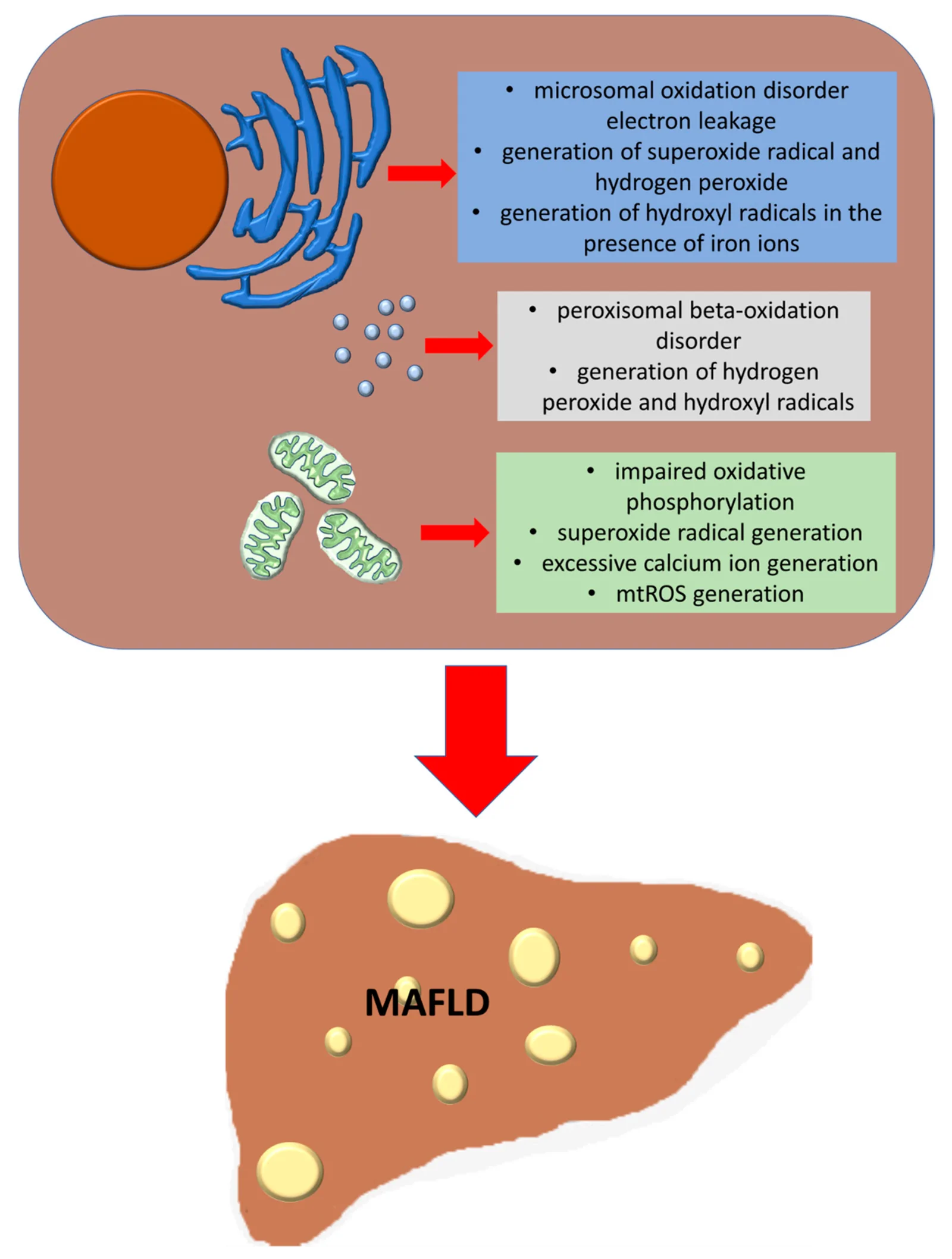
Key disturbances in hepatocytes leading to excessive generation of ROS and MAFLD.

Figure 1 shows the key disturbances in hepatocytes that lead to excessive formation of reactive oxygen species and non-alcoholic fatty liver disease.

In the ER, microsomal oxidation is disrupted, resulting in the activation of oxygen molecules before they bind to substrates. Oxygen is reduced not to water, but to reactive ROS such as superoxide anions and hydrogen peroxide. In the presence of ferrous iron ions, hydrogen peroxide can convert to the more toxic hydroxyl radical. When oxidative phosphorylation in mitochondria is impaired, superoxide anions are formed as by-products, and excess mitochondrial Ca^2+^ can also significantly contribute to the formation of mtROS, which ultimately contributes to mtDNA damage, which also accelerates the development of MAFLD. Hydrogen peroxide can also be formed during peroxisomal β-oxidation, subsequently converting into a hydroxyl radical.

### 2.2. Antioxidant and Other Functions of Selenoproteins in MAFLD

The role of selenoproteins in MAFLD (MAFLD) is actively being studied. It has been established that 14 selenoprotein genes (SELENOF, SELENOI, SELENOK, SELENOO, SELENOS, SELENOV, SELENOT, SEPHS2, TXNRD2, TXNRD3, GPX1, GPX2, MRSB1, and DIO2) had lower expression in MAFLD [75]. Another study found that all genes with higher expression in steatosis also had higher expression in MAFLD (SELENOF, SELENOK, SELENOS, SELENOT, SELENOW, GPX2, GPX3, DIO1, and SEPHS2), in addition to the TXNRD1 gene [76]. Similarly, four of the five genes with lower expression in steatosis also had lower expression in MAFLD (SELENOO, SELENON, DIO3, and TXNRD3). Four selenoprotein genes were also shown to have differential expression in MAFLD: one with lower expression (SELENOP) and three with higher expression (SELENON, GPX1, and GPX4) in MAFLD [77]. However, the functions of most of these selenoproteins remain poorly understood.

One of the most abundant selenoproteins that provides basic antioxidant protection to the liver is GPX1. GPX1 deficiency in hepatocytes was found to increase hydrogen peroxide generation, but its levels did not contribute to liver or systemic stress in mice. Furthermore, GPX1 deficiency was not associated with non-alcoholic steatohepatitis (NASH), which is characterized by fatty liver disease and inflammation that can lead to steatotic hepatocyte death and fibrosis [78,79]. However, GPX1 deficiency contributed to a decrease in lymphocytic infiltration in the liver and suppression of hepatic inflammation, contributing to a decrease in fibrosis and a decrease in the expression of inflammatory markers and fibrogenesis [80]. One study demonstrated that carriers of the GPX1 gene promoter genotypes C594T (CT) and C594T (TT) may have a high risk of developing MAFLD [81]. However, another study did not confirm the association between GPX1 genetic variants and a predisposition to MAFLD [82]. The explanation for why GPX1 (the main hydrogen peroxide scavenger) deficiency does not exacerbate liver damage, but rather protects against inflammation and fibrosis, is presumably linked to the compensatory action of other cellular peroxidases. Furthermore, at low and moderate concentrations, hydrogen peroxide does not cause oxidative stress, but rather activates the major cell-protective transcription factor NRF2, enhancing insulin signaling in the liver, which protects cells from metabolic collapse [83,84]. Complete absence of GPX1 alters the intracellular glutathione pool and H_2_O_2_ kinetics, which disrupts the classical NF-κB signaling cascade. This results in the liver losing its ability to generate chemokines for immune cells, so lymphocytes circulate in the bloodstream but are unable to migrate into the organ parenchyma [85].

It is well known that the liver is a major organ for iron storage and lipid metabolism, and ferroptosis plays a critical role in the pathological progression of MAFLD [86,87,88]. The main biochemical changes in ferroptosis are glutathione (γ-glutamylcysteinylglycine, GSH) depletion and decreased glutathione peroxidase 4 (GPX4) activity [89]. Glutathione is a cofactor of GPX4, which is able to remove lipid hydroperoxides in biological membranes and use glutathione to reduce lipid peroxides [90,91]. A study was also conducted that demonstrated that the decrease in the level of the cytoplasmic isoform of GPX4 (cGPX4) was not different in patients with MAFLD and healthy individuals, but in patients with this disease and in model mice, overexpression (3.5 times higher than in controls) of the nuclear form of GPX4 (nGPX4) was observed [92]. The authors found that cGPX4 successfully inhibited not only the increased level of iron ions, but also suppressed the expression of proferroptotic proteins: ACSL4, ALOX15, ferritin, transferrin, 3-nitrotyrosine, 4-HNE, TFR1 and ZIP14. Whereas the second isoform, on the contrary, promoted ferroptosis, aggravated glutathione/glutamine depletion, fibrosis, and liver inflammation in mice with MAFLD. Thus, the authors demonstrated that the two GPX4 isoforms have opposing effects on ROS formation and ferroptosis in hepatocytes [92].

It is well known that, in addition to the glutaredoxin system, there is a major thiol-dependent antioxidant system—the thioredoxin (Trx) system, represented by Trx, thioredoxin reductase (TXNRD), and NADPH [93,94,95]. Since TXNRD accepts electrons from NADPH and transfers them to Trx, the proper functioning of the thioredoxin system largely depends on TXNRD activity. Thus, a number of studies have shown that in adipose tissue with metabolic syndrome, there is a decrease in TXNRD1 expression and a simultaneous increase in the level of the oxidized form of Trx, which contributes to increased oxidative stress due to the consumption of saturated fats [96,97,98,99]. It was also found that in non-alcoholic steatohepatitis, which is a dangerous progressive stage of MAFLD, a decrease in the expression of TXNRD1 and TXNRD2, but not TXNRD3, was observed [100]. In contrast, in mice resistant to steatohepatitis, activation of the TXNRD1 gene in the liver was observed [101,102].

The main Se transporter protein, which is predominantly synthesized by hepatocytes, is SELENOP. It has been established that this selenoprotein can play a dual role in MAFLD: on the one hand, it acts as an antioxidant; on the other hand, it contributes to the development of insulin resistance and systemic metabolic disorders [103,104,105]. Numerous studies of the role of SELENOP in MAFLD demonstrate that the level of this selenoprotein decreases during acute inflammation and increases during long-term obesity and chronic inflammation [106,107,108,109]. When SELENOP synthesis is impaired in mice, hyperleptinemia, hyperinsulinemia, insulin resistance, hepatic steatosis, and increased oxidative stress in the liver were observed. When SELENOP synthesis is impaired in mice, hyperleptinemia, hyperinsulinemia, insulin resistance, hepatic steatosis, and increased oxidative stress in the liver were observed [108,109]. However, there is a number of conflicting data indicating high levels of Se and SELENOP in MAFLD [104,105]. These contradictory data can be explained by the fact that SELENOP is located at the intersection of two opposing processes: the cellular protective antioxidant response and the body’s pathological metabolic response to excess glucose and fat. Since chronic obesity and MAFLD lead to the development of liver insulin resistance, this inhibits SELENOP synthesis. In a healthy liver, insulin is a potent suppressor (brake) of SELENOP expression. During acute inflammation, the rise in pro-inflammatory markers blocks its translation, urgently redirecting selenium to the synthesis of intracellular antioxidants (e.g., GPX1) to protect the cell from death [105,106,107,108]. It can be hypothesized that the decreased SELENOP levels in MAFLD may be related to the fact that, since SELENOP is a complex, highly glycosylated protein containing up to 10 selenocysteine residues, its folding in the ER is an energy-consuming process. When hepatocytes become overloaded with free fatty acids, ER membrane fluidity is impaired. Severe ER stress develops as a result of chaperone malfunction, leading to SELENOP misfolding and subsequent degradation. Therefore, the available data are not yet sufficient to use SELENOP for the diagnosis or treatment of MAFLD.

SELENOM, a key thioredoxin-like enzyme present in the ER, is associated with hepatocyte degeneration and carcinoma, and stable overexpression of SELENOM prevents hydrogen peroxide-induced oxidative cell damage [110,111,112]. SELENOM affects multiple pathways, including redox balance control, fatty acid composition, stress response, and cancer cell proliferation [27,28,29]. SELENOM levels were shown to be decreased in MAFLD, and SELENOM knockout animals had increased susceptibility to high-fat diet-induced fatty liver disease in mice [110]. Inhibition of SELENOM activity contributed to high-fat diet-mediated hepatic alveolar steatosis and fibrosis, increased inflammatory response, and oxidative stress in the liver. The increase in oxidative stress was due to the fact that a high-fat diet itself causes a decrease in the levels of key antioxidants in the liver (CAT, GPX, SOD), and knockdown of SELENOM further reduces their expression, while the level of MDA, on the contrary, increased [20]. In addition, SELENOM deficiency caused a decrease in fatty acid oxidation and increased lipid deposition in mice, and weakened mitophagy activity in hepatocytes, which contributed to mitochondrial apoptosis. In turn, overexpression of SELENOM in hepatocytes reduced the level of mitochondrial ROS, activated mitophagy by increasing Parkin expression through the AMPKα1-MFN2 pathway, which is involved in maintaining the biological function of mitochondria and regulates the process of energy metabolism [111,112]. Furthermore, the AMPKα1-MFN2 signaling pathway involves increased expression of proinflammatory markers involved in MAFLD [113].

Another ER selenoprotein is SELENOS, which is actively involved in the regulation of ER stress and plays an important role in obesity, insulin resistance, glucose and lipid metabolism [114,115,116,117,118]. MAFLD is known to be closely associated with insulin resistance and type 2 diabetes mellitus, and SELENOS is also closely associated with these diseases. When analyzing the expression of this selenoprotein in the liver of mice fed a high-fat diet, as well as in mice with type 2 diabetes, it was found that the expression levels of this selenoprotein were reduced in all models. This contributed to the enhancement of ER stress markers, the progression of steatosis due to increased absorption of fatty acids and a decrease in their oxidation [118]. A sharp increase in ER stress in hepatocytes contributed to changes in fatty acid absorption, lipid synthesis, and fatty acid oxidation. Furthermore, SELENOS knockout mice exhibited decreased production of the hepatokine FGF21 and the adipokine adiponectin, as well as increased adipose tissue size. Liver-specific deletion of the SELENOS gene also contributed to the development of insulin resistance, impaired glucose metabolism, and activation of hepatic protein kinase Cε (PKCε).

Another selenoprotein highly expressed in the liver is SELENOW, which has antioxidant activity and regulates the redox status of macrophages during inflammation [118,119,120]. In a study investigating the role of SELENOW in MAFLD, it was found that this selenoprotein is significantly elevated in the liver of patients with this disease [121]. The authors showed that this selenoprotein damages mitochondria, induces mtROS generation, and mediates mitochondrial apoptosis via pyruvate kinase M2 (PKM2) to trigger HIF-1α transactivation. PKM2 is known to be a coactivator for HIF1α binding after nuclear translocation [122]; therefore, SELENOW mediates mitochondrial apoptosis by acting on PKM2 to trigger HIF-1α activation. Furthermore, SELENOW overexpression is associated with activation of pyroptosis and inflammasome formation in MAFLD, which promotes mtDNA leakage and leads to activation of the innate immune system component cGAS-STING (cyclic GMP-AMP synthase—stimulator of interferon genes) signaling pathway in macrophages and macrophage polarization [121].

Relatively recently, it was shown that hypothyroidism and thyroid hormone receptor β mutations increase the risk of MAFLD, and thyroid hormones and their analogs are effective in the treatment of MAFLD comorbidities [123,124,125,126]. It is known that the levels of the prohormone thyroxine and its active form T3 in the liver are regulated by deiodinase enzymes (DIOs), of which there are three, and all of them contain selenocysteine. On the one hand, it was shown that the DIO1 level decreases in the late stage of MAFLD [79], whereas the activity of the DIO1 gene increases in hyposteatosis and the early stage of MAFLD, which is necessary for maintaining the intrachain concentration of T3 [127]. With a decrease in the expression and activity of DIO1 in the liver, a decrease in the metabolism of intrahepatic T4 to T3, an increase in the synthesis of fatty acids, and a decrease in the β-oxidation of fatty acids were observed. Furthermore, DIO1 knockdown mice exhibited significant expression of inflammatory and fibrotic markers [127]. Another study demonstrated that thyroid hormone dysregulation in the liver increases susceptibility to MASLD in the elderly. Specifically, senescent hepatocytes displayed phenotypes of the senescent liver, with decreased DIO1 mRNA expression and increased DIO3 mRNA expression compared to controls [128]. Furthermore, immunohistological analysis revealed that the mild MAFLD group had lower DIO1 staining intensity than controls, with some patients even exhibiting rare parenchymal areas completely devoid of DIO1 [129]. In turn, DIO3 staining intensity increased with fibrosis progression in MAFLD patients, with a strong sinusoidal accumulation of DIO3 observed. Such changes in DIO1 and DIO3 expression and activity were associated with worsening intrahepatic hypothyroidism due to decreased expression of the T3-responsive gene THRSP in the liver of MAFLD patients [129]. Thus, patients with low DIO1 expression may be at higher risk of developing hepatosteatosis and MAFLD.

SELENOH is a selenoprotein that, along with SELENOT, SELENOV, and SELENOW, belongs to the family of thioredoxin-like proteins. It has been found to possess glutathione peroxidase-like activity toward hydrogen peroxide [130]. SELENOH has been found to be a key regulator of fatty acid oxidation in the liver through its interaction with the peroxisome proliferator-activated receptor alpha (PPARα). SELENOH has been found to be a key hepatoprotective selenoprotein regulating fatty acid oxidation in the liver [131]. It is well established that PPARα activation enhances fatty acid oxidation and can improve lipid and bile acid homeostasis in bile; PPARδ/β maintains metabolic flexibility and anti-inflammatory polarization of macrophages; and PPARγ helps maintain hepatic stellate cells in a quiescent state with less fibrogenicity. The clinical and experimental benefits observed with PPAR agonists in cholestatic liver diseases support the hypothesis that similar modulation of the metabolic-inflammatory-oxidative axis may inhibit fibrosis progression in NAFLD [132]. Thus, SELENOH deficiency in the liver contributed to extensive fibrosis and inflammation of the liver, and a decrease in the expression of genes regulating fatty acid oxidation steps in both mitochondria and peroxisomes. While overexpression of SELENOH in hepatocytes contributed to a clear improvement in the pathology of steatohepatitis, one of the progressive stages of MAFLD, the authors suggested that mice deficient in SELENOH exhibited increased liver triglyceride levels during fasting, most likely due to decreased activity of fatty acid oxidation via PPARα-mediated hydrogen bonding and salt bridges [133].

**Figure 2:**
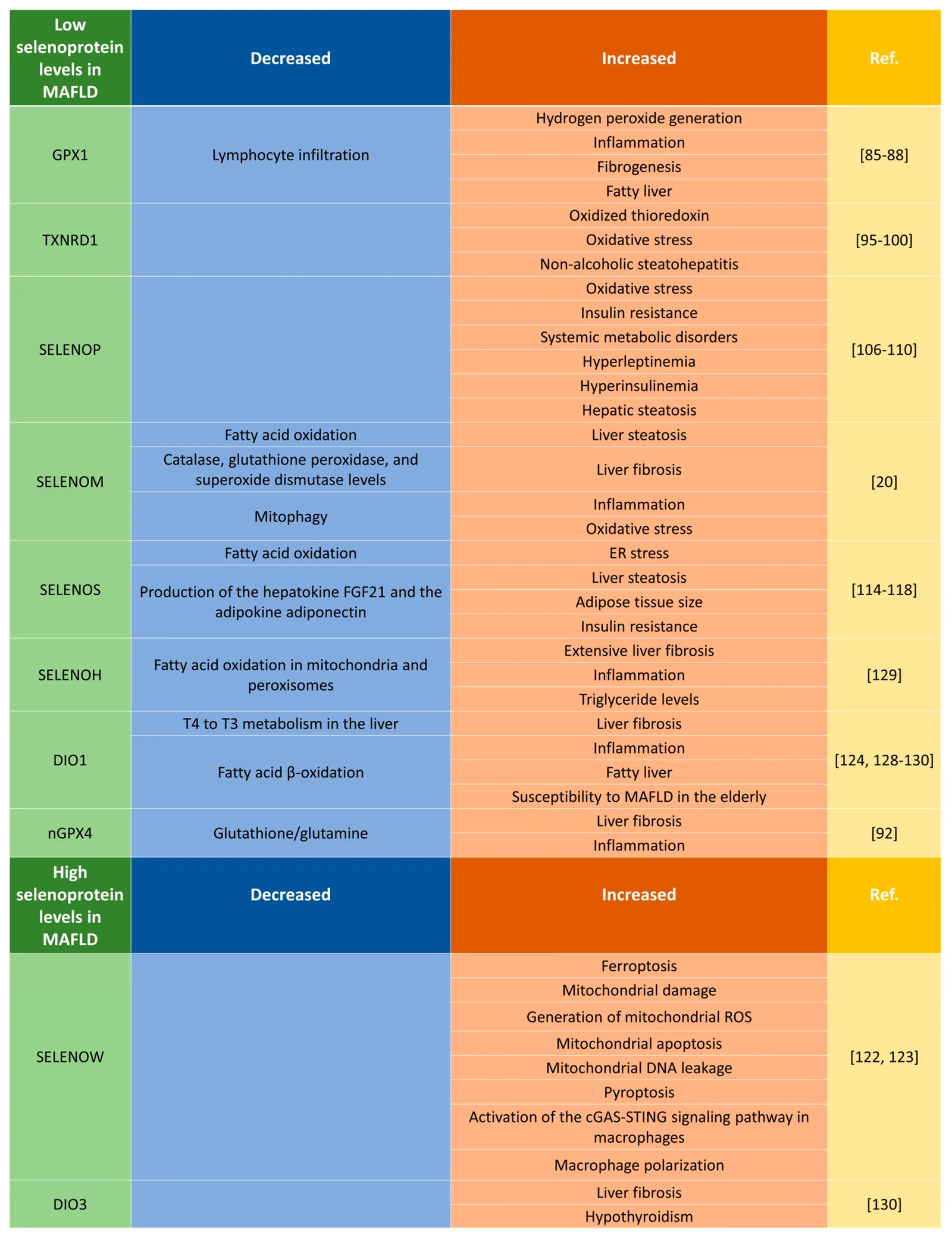
Pathological processes in the liver caused by changes in selenoprotein levels, leading to the development of MAFLD.

Thus, the currently available information regarding the role of selenoproteins in MAFLD suggests that seven of the 10 selenoproteins (GPX1, TXNRD1, TXNRD2, SELENOP, SELENOM, SELENOH, and DIO1) have been shown to have decreased activity and expression in MAFLD. Three of these (the nuclear form GPX4, SELENOW, and DIO3), on the contrary, are characterized by high expression in MAFLD. The main functions of these selenoproteins in MAFLD are schematically shown in Figure 2.

Selenoproteins whose expression in the liver is increased or decreased are separated. Consequences that depend on the level of selenoprotein expression are color-coded: if certain processes are enhanced (orange), if certain processes are suppressed or inhibited (blue). FGF21—fibroblast growth factor 21; T3—triiodothyronine; T4—thyroxine; cGAS—cyclic GMP-AMP synthase; STING—stimulator of interferon genes. 

## 3. Alcoholic Liver Disease

Alcoholic liver disease (ALD) is a major cause of acute and chronic liver injury. Over the past decades, extensive data have accumulated on the pathological process of ALD. The term ALD encompasses a range of disorders, including simple steatosis, steatohepatitis, cirrhosis, and end-stage hepatocellular carcinoma (HCC). The development of ALD is caused by disruption of hepatic metabolic homeostasis following intestinal alcohol absorption and subsequent metabolism in the liver, which is typically accompanied by lipid accumulation in hepatocytes and leads to the development of hepatic steatosis [134,135]. Furthermore, 8–20% of patients with ALD develop cirrhosis, and 3–10% develop HCC [136].

### 3.1. The Role of ROS in the Development of Alcohol-Induced Liver Disease

Ethanol oxidation by alcohol dehydrogenase (ADH) in the cytosol of hepatocytes occurs with the reduction of the coenzyme NAD^+^ to NADH. NADPH is not involved in this reaction. An alternative pathway for alcohol metabolism occurs in the ER via the microsomal ethanol oxidation system. Cytochrome P4502E1 catalyzes the oxidation of ethanol to acetaldehyde, using NADPH as an electron donor. This process oxidizes NADPH to NADP+, producing superoxide anion, peroxide, and other ROS [137,138,139,140,141,142,143,144,145,146]. Additionally, as in MAFLD, alcohol metabolism can cause ER stress, which is accompanied by disruption of Ca^2+^ homeostasis and subsequent mtROS formation. Furthermore, chronic alcohol exposure to the liver leads to impaired glutathione transport from the cytosol to the mitochondria in hepatocytes. Glutathione transport is a key protector of mitochondria from oxidative stress in the absence of catalase in this organelle. Depletion of glutathione in mitochondria is accompanied by decreased glutathione synthase activity, which increases hepatocyte sensitivity to TNF-α and exacerbates liver damage [147,148,149].

Another important source of oxidative stress in ALD is the activity of NADPH oxidase and iNOS in Kupffer cells [150,151]. Furthermore, alcohol impairs the breakdown of oxidized proteins in proteasomes, which can be accompanied by an increase in TNF-α and cause oxidative stress in the mitochondria of hepatocytes [152,153]. Alcohol also causes apoptotic death of hepatocytes, as chronic alcohol consumption has been shown to promote the growth of apoptotic bodies in hepatocytes [154].

### 3.2. Antioxidant and Other Functions of Selenoproteins in ALD

Acute ethanol administration is known to alter Se homeostasis and distribution in the body, affect its biological activity, and contribute to oxidative liver injury. Se is an effective cofactor for glutathione peroxidase and has an antioxidant effect, which improves the condition in ALD. Furthermore, in patients with ALD, ethanol-induced oxidative damage stimulates the expression of selenoproteins such as GPX1, GPX4, and SELENOP, which reduces ROS levels and attenuates oxidative stress, inflammation, lipid metabolism disorders, and lipid peroxidation, thereby contributing to the alleviation of ALD symptoms [155]. A direct, clear relationship between Se levels and GPX3 activity in the serum of adolescent rats suffering from binge drinking was also established [156]. In addition, this group exhibited lower GPX1 expression and activity, as well as Se depletion in the liver, which causes lipid and protein oxidation. The same study demonstrated that GPX4 and NFkB expression patterns change in direct proportion in adolescent rats subjected to binge alcohol consumption. Serum SELENOP expression was also significantly reduced, along with GPX3, in these animals.

However, another study showed that moderate alcohol consumption, on the contrary, resulted in an increase in serum SELENOP levels, independent of liver enzyme levels and fat content [157]. It is well known that SELENOP is a key redox protein in the body, and its excess can lead to reductive stress [158]. A study of the mechanism by which ethanol enhances SELENOP synthesis in hepatocytes found that this occurs through increased nuclear translocation of FoxO3a via activation of the ERK-FoxO3a pathway [159]. Using the HepG2 cell line as an example, it was shown that SELENOP expression was induced by ethanol, rather than acetaldehyde or acetic acid. In turn, the authors found that the level of cytosolic FoxO3a protein decreased upon ethanol stimulation, while the level of nuclear FoxO3a, conversely, increased, which was also accompanied by phosphorylation of ERK and an increase in DUSP5 and DUSP6 phosphatases. Thus, the authors demonstrated that 2% ethanol promoted increased SELENOP expression in HepG2 cells, which was associated with the AMPK-FoxO3a signal [159]. Thus, to date, data on the effect of alcohol consumption on Se and SELENOP appear contradictory.

To explain the contradictory data on the effect of alcohol on SELENOP levels, it can be hypothesized that moderate concentrations of unbound ethanol act as a regulatory stimulus, activating the transcription factor FoxO3a via the ERK/DUSP/AMPK cascades, leading to a compensatory increase in SELENOP secretion by hepatocytes. Conversely, with chronic abuse, the toxic effect of acetaldehyde plays a leading role, inducing steatosis and ER stress, mechanically and functionally blocking the liver’s secretory apparatus. This clinically manifests as a sharp drop in serum selenoprotein levels (SELENOP and GPX3) against the background of general depletion of the selenium pool.

**Figure 3:**
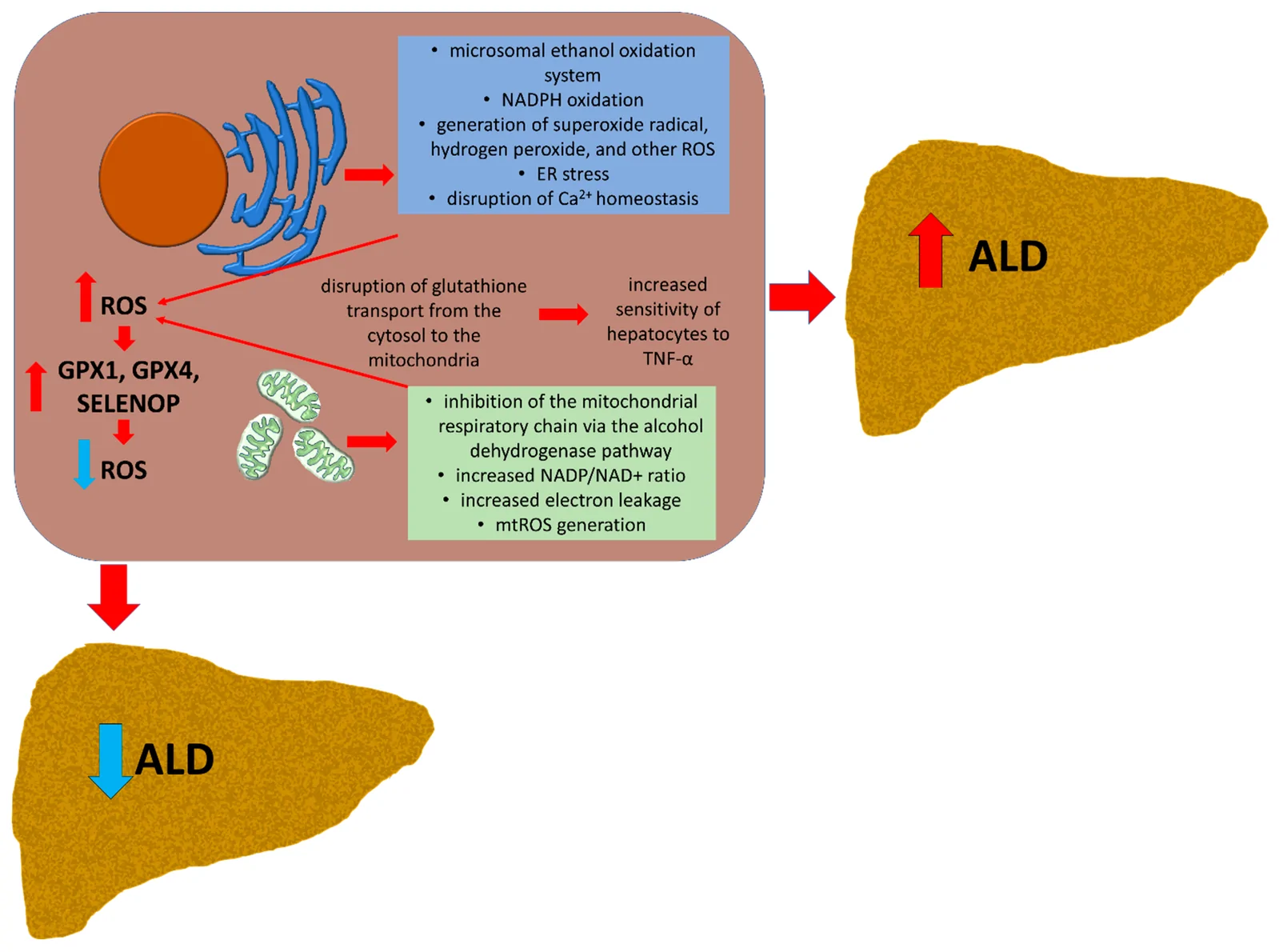
Key disturbances in hepatocytes leading to excessive generation of ROS and ALD.

Figure 3 shows key disturbances in hepatocytes leading to excessive generation of ROS and ALD.

Ethanol metabolism results in inhibition of the mitochondrial respiratory chain, increased electron leakage, and ROS generation. An alternative pathway for alcohol metabolism occurs in the ER via the microsomal ethanol oxidation system. Cytochrome P4502E1 (CYP2E1) catalyzes the oxidation of ethanol to acetaldehyde, using NADPH as an electron donor. This process generates superoxide anion, peroxide, and other ROS. Alcohol can induce ER stress, which is accompanied by disruption of Ca^2+^ homeostasis and subsequent mtROS formation. Chronic alcohol exposure to the liver disrupts glutathione transport from the cytosol to the mitochondria in hepatocytes. Glutathione is a key protector of mitochondria from oxidative stress in the absence of catalase in this organelle. Depletion of glutathione in mitochondria is accompanied by a decrease in glutathione synthase activity, which increases the sensitivity of hepatocytes to TNF-α and increases liver damage. The increase in ROS in the liver is accompanied by an increase in the expression of selenoproteins GPX1, GPX4, and SELENOP, which reduce the level of ROS, oxidative stress, inflammatory response, lipid metabolism disorders, and lipid peroxidation, thereby helping to alleviate the symptoms of ALD.

## 4. Hepatitis

Hepatitis is an inflammatory liver disease caused by various exogenous and endogenous factors, the severity of which can range from mild and self-limiting to severe, including liver fibrosis, cirrhosis, cancer, and/or liver failure. Viral hepatitis is mainly caused by hepatotropic hepatitis viruses A, B, C, D, or E (abbreviated as HAV, HBV, HCV, HDV, and HEV, respectively) [160,161,162]. Hepatic steatosis is known to be a common histological feature of chronic hepatitis C and is more often associated with HCV genotype 3 [163]. In patients with HCV, the prevalence of steatosis ranges from 40% to 86% (mean ~55%) [164,165,166], and “viral fat” exists in patients with HCV genotype 3 (especially in patients with genotype 3). The mechanisms underlying this genotype-specific steatosis are unknown. Existing studies suggest that HCV can inhibit the secretion of very low-density lipoproteins (LDL), which is corrected by antiviral therapy. Studies of the molecular mechanisms of such regulation have established that the HCV core protein is sufficient to induce lipid accumulation in hepatocytes, but the role of NS5A (Nonstructural protein 5A) appears to be limited to cells expressing the HCV core protein. These proteins interact with apolipoproteins A1 and A2, which are likely involved in the accumulation and storage of triglycerides in hepatocytes. Furthermore, the HCV core protein inhibits the activity of microsomal triglyceride transfer protein (MTP), which is accompanied by the accumulation of non-secreted triglycerides and steatosis [167].

### 4.1. The Role of ROS in Hepatitis Development

Oxidative stress is now well known to play an important role in the progression of hepatitis, as hepatitis viruses are capable of activating oxidative stress and ROS-sensitive signaling pathways [168,169]. It is well known that hepatitis viruses can cause mitochondrial dysfunction by inhibiting the mitochondrial electron transport chain and reducing intracellular and mitochondrial glutathione levels [170,171]. Levels of ROS, lipid peroxidation, and leukocyte thioredoxin-8-OH-deoxyguanosine were elevated in the serum of patients with hepatitis C [172,173,174]. Elevated NADPH oxidase in hepatitis C contributes to increased generation of ROS and reactive nitrogen species. Furthermore, patients with chronic hepatitis C often have increased production of TNF-α, which causes oxidative stress by stimulating the generation of ROS such as the superoxide radical and hydrogen peroxide. It is well known that HCV infection, via core proteins and Nonstructural protein 3 (NS3), can induce NO, which is capable of not only introducing double-strand breaks into DNA but also inactivating enzymes of the mitochondrial respiratory chain [175,176,177,178,179]. This, in turn, causes a blockage of the electron transport chain, disruption of the mitochondrial transmembrane potential, and electron leakage, which contributes to the generation of endogenous ROS [175].

It has also been established that HCV infection can constitutively activate STAT3, leading to its nuclear translocation, which is mediated by core proteins, E1 and NS3, and ROS [175]. Since STAT3 is an oncogene and a major inducer of acute-phase liver disease pathology, this has negative consequences. In liver cells replicating the hepatitis C virus, the redox potential was increased, which was partly due to impaired catalase activity [176].

Furthermore, it is well known that ROS levels are often elevated in the liver and blood of patients with the hepatitis B virus (HBV). HBV itself, during infection, induces ROS generation through the HBx protein, which interacts with the voltage-dependent anion channel in mitochondria, altering its transmembrane potential. This is accompanied by a decrease in the activity of mitochondrial enzymes involved in electron transfer and an increase in mtROS [180,181,182,183]. It was also found that HBx requires p53 transcriptional activity to increase ROS levels. Furthermore, HBx reduced catalase levels but increased Mn-SOD and GPX levels, which also contributed to an increase in p53 and ROS levels [184]. In hepatitis caused by HBV and HCV viruses, viral proteins accumulate in the ER, triggering calcium leakage, which disrupts the mitochondrial electron transport chain and causes an increase in ROS. Increased ROS generation typically activates the Nrf2, NF-kB, and MAPK signaling pathways [185]. Chronic hepatitis also increases oxygen consumption, which leads to the release and accumulation of ROS at the site of injury, triggering a cycle of inflammation and oxidative stress.

### 4.2. Antioxidant and Other Functions of Selenoproteins in Hepatitis

Serum Se concentrations in patients with hepatitis B and C were 101.60 ± 0.55 and 77.43 ± 0.47 mg/L in men and women with hepatitis C, respectively, while in patients with hepatitis B, the concentrations were 107.58 ± 0.44 and 137.8 ± 0.36 mg/L in men and women, respectively. Se concentrations in healthy individuals ranged from 30 to 580 mg/L, of which 90–470 mg/L were found in women and 30–580 mg/L in men [186]. SELENOP mRNA expression has been found to be elevated in the liver of patients with chronic hepatitis C due to increased expression of the alpha subunit of CCAAT/enhancer-binding protein (CEBPα), which is an essential transcription factor and activates the SELENOP promoter [187]. SELENOP mRNA directly binds to retinoic-acid-inducible gene I (RIG-I) and inhibits the RIG-I-mediated response to type I interferon. Using FISH and immunofluorescence staining, SELENOP mRNA and RIG-I were shown to colocalize in HepG2 mRNA cells, with mRNA, but not protein, playing an important role in inhibiting RIG-I activity, which also affects HCV replication and HCVcc infection in several cell lines, including KH cells, HepG2 cells, and PHH cells. These data suggest that mRNA may play a role in immune tolerance by inhibiting RIG-I activity in the liver [187]. HCV may exploit this tolerance system, and increased expression of SELENOP mRNA would be beneficial for establishing persistent infection. Furthermore, recent studies have shown that RIG-I acts as an innate sensor and direct antiviral factor for hepatitis B virus [188], suggesting a possible involvement of SELENOP mRNA in hepatitis B virus replication. Another study investigated the involvement of SELENOP in hepatitis B virus X protein (HBx)-induced lipid peroxidation in the HepG2 cell line [189]. It was found that HBx overexpression reduced SELENOP expression, while lipid peroxidation was increased by 2.5-fold and there was a 3- to 6-fold increase in TNF-α mRNA and protein expression compared to the control. These results further highlight the fact that SELENOP is an antioxidant selenoprotein that protects cells, particularly hepatocytes, from POL.

A relationship was established between chronic hepatitis C, activation of POL, and decreased GPX4 activity, which was facilitated by the GPX4 gene polymorphism (718C/T) [190]. In addition, it was found that the levels of GPX4 expression in peripheral mononuclear cells and serum of patients with chronic hepatitis B were lower than in healthy individuals, while the methylation levels of the GPX4 promoter were, on the contrary, higher [191]. It is well known that GPX4 can regulate the cGAS-STING pathway, which plays an important role in the immune response, since cGAS is a receptor for the recognition of abnormal DNA produced by viruses and bacteria; after recognition, it can induce the synthesis of cyclic GMP-AMP (cGAMP) and activate the downstream STING (endoplasmic reticulum-associated dimer protein) [192,193,194,195]. The authors found that GPX4 deficiency caused by promoter methylation enhances lipid peroxidation and specifically inhibits the cGAS-STING pathway in HBV patients, creating a state of immune tolerance [191]. In another study, it was demonstrated that GPX4-regulated ferroptosis mediates experimental autoimmune hepatitis induced by S100 and is associated with the NRF2/HO-1 pathway [196]. Autoimmune hepatitis is a disease characterized by inflammation, the presence of autoantibodies, hypergammaglobulinemia, and interface hepatitis, which is often studied using a mouse model of AIH induced by the liver-specific antigen S100 [197,198,199]. The authors found that suppression of GPX4 expression leads to increased ALT and AST levels in serum, an increase in lipid peroxidation, an increase in MDA, and iron overload in the liver of mice with autoimmune hepatitis. Thus, it was confirmed that GPX4 is vital for preventing the consequences of lipid peroxidation and ferroptosis in autoimmune hepatitis.

**Figure 4:**
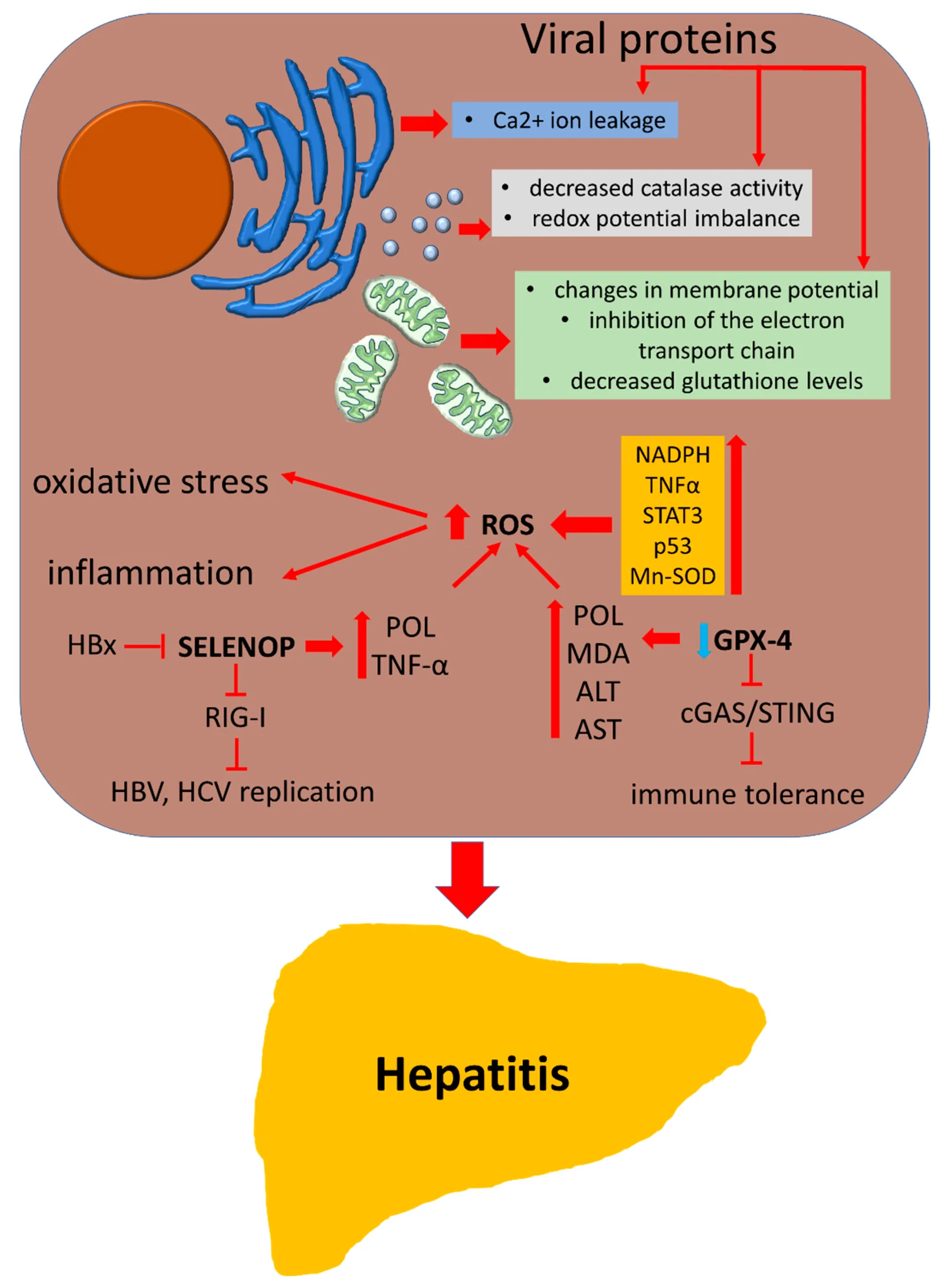
Pathogenesis of hepatitis caused by viral proteins.

Pathogenesis of hepatitis caused by viral proteins is shown schematically in Figure 4.

Viral proteins affect three key cellular structures: the ER, causing Ca^2+^ leakage into the cytosol; peroxisomes, reducing catalase activity and disrupting the redox potential; and mitochondria, altering membrane potential, inhibiting the electron transport chain, and reducing glutathione levels. These disruptions lead to a sharp increase in ROS, which activates inflammatory and oxidative stress factors, and also increases the expression of proteins and cytokines: NADPH, TNFα, STAT3, p53, and Mn-SOD. The viral protein HBx suppresses the SELENOP protein, reducing SELENOP’s blocking effect on the RIG-I receptor, which normally inhibits viral replication. This results in activation of HBV and HCV replication, increased lipid peroxidation, and increased TNF-α expression. Furthermore, increased ROS leads to decreased GPX4 activity, which triggers the activation of liver damage biomarkers, POL, MDA, ALT, and AST. Decreased GPX4 also weakens the cGAS/STING pathway, leading to the development of immune tolerance, which hinders the body’s ability to fight infection. The combination of these destructive processes (oxidative stress, cell death, inflammation, and accelerated viral replication) leads to the development of chronic liver inflammation—hepatitis.

## 5. Liver Fibrosis and Cirrhosis

Fibrosis is a pathological process in which functional liver cells (hepatocytes) are gradually replaced by scar tissue in response to chronic damage or inflammation. Without timely treatment, this leads to impaired blood circulation and liver function [200,201,202]. Over time, these pathological processes lead to irreversible fibrosis—liver cirrhosis—where normal liver tissue is completely replaced by scar tissue, forming regenerative nodules that compress blood vessels and bile ducts. This irreversible disruption of liver structure leads to the gradual failure of all liver functions [203,204,205,206,207]. Patients with advanced liver fibrosis are known to exhibit a distinct microbial signature independent of the etiology of the liver disease and other comorbidities. These results suggest that microbes and their metabolites contribute to the development of liver fibrosis, with patients with advanced fibrosis exhibiting a distinct microbiome signature compared to those with earlier stages of fibrosis. These differences are characterized by an increase in the number of Prevotella bacteria and a decrease in the number of Bacteroides bacteria [208].

### 5.1. The Role of ROS in the Development of Liver Fibrosis and Cirrhosis

Oxidative stress is a central mechanism in the pathogenesis of liver fibrosis and cirrhosis, enhancing inflammation and fibrogenesis in the liver. Thus, as liver fibrosis progresses, ethanol can increase the generation of ROS and reactive nitrogen species (RNS), which trigger proinflammatory cytokines and markers of fibrogenesis [209,210]. Thus, liver fibrosis arises from a long-term imbalance between ROS formation and the ability of antioxidants to neutralize these ROS, which act as metabolic byproducts and produce lipid peroxidation, protein oxidation, and activation of hepatic stellate cells. As a result, stellate cells transdifferentiate into myofibroblasts and deposit extracellular matrix proteins, including type I collagen, leading to fibrotic scarring [211,212]. Regardless of the etiology of these liver pathologies, they are all characterized by an oxidative environment that exacerbates hepatocyte damage and promotes the progression of fibrosis and cirrhosis.

In liver fibrosis, specific POL markers are elevated, which directly trigger the activation of hepatic stellate cells and the expression of collagen genes [213]. This, in turn, leads to disruption of mitochondrial autophagy and mtDNA production and mitochondrial damage-associated molecular patterns (mDAMPs). All these events trigger an autocrine inflammatory response in neighboring hepatocytes and Kupffer cells, resulting in mitochondrial damage. Thus, ROS-mediated mDAMPs play an important role in stimulating stellate cell proliferation, their differentiation into myofibroblasts, and collagen secretion, which causes liver fibrosis [214,215].

### 5.2. Antioxidant and Other Functions of Selenoproteins in Liver Fibrosis and Cirrhosis

We have previously shown this at the mRNA and protein levels in fibrosis induced by intraperitoneal injections of thioacetamide in mice. We found that liver fibrosis significantly increased the levels of three glutathione peroxidases (GPX2, GPX3, and GPX4), while the levels of three thioredoxin reductases and three deiodinases did not differ significantly from controls. We also found that liver fibrosis significantly increased the expression of other selenoproteins: SELENOM, SELENOS, SELENON, and SELENOW. However, mRNA expression of SELENOI and SELENOP decreased. However, in TAA-induced cirrhosis, it was found that thioredoxin reductase activity in animal serum increased, while glutathione peroxidase activity in animal serum, on the contrary, decreased, which largely depended on selenium supplementation [216]. It was previously shown that in cirrhosis, the plasma concentration of SELENOP increases with the progression of the disease [217]. It was also found that GPX3 activity increased with the progression of liver cirrhosis (150% relative to the control), which may be caused by the release of intracellular isoforms of the enzyme from the affected liver or other tissues [218]. It was established that excessive iron deposition in the liver and ferroptosis can increase liver fibrosis; in addition, iron is contained in large quantities in hepatic stellate cells [219,220,221]. Among the 25 mammalian selenoproteins known to date, GPX4 is a key participant in ferroptosis [222,223,224,225,226]. One of the known signaling pathways, the activation of which significantly reduces ferroptosis levels in liver fibrosis, is NRF2-GPX4 [226,227,228]. GPX4 inhibits ferroptosis induced by lipid peroxidation and is a target of the essential transcription factor NRF2. Altered thyroid hormone homeostasis with reduced levels of fT3 and similar levels of fT4 has been described in cirrhosis and defined as “low fT3 syndrome” [229,230]; however, studies devoted to the direct role of deiodinases in these processes have not been found to date.

**Figure 5:**
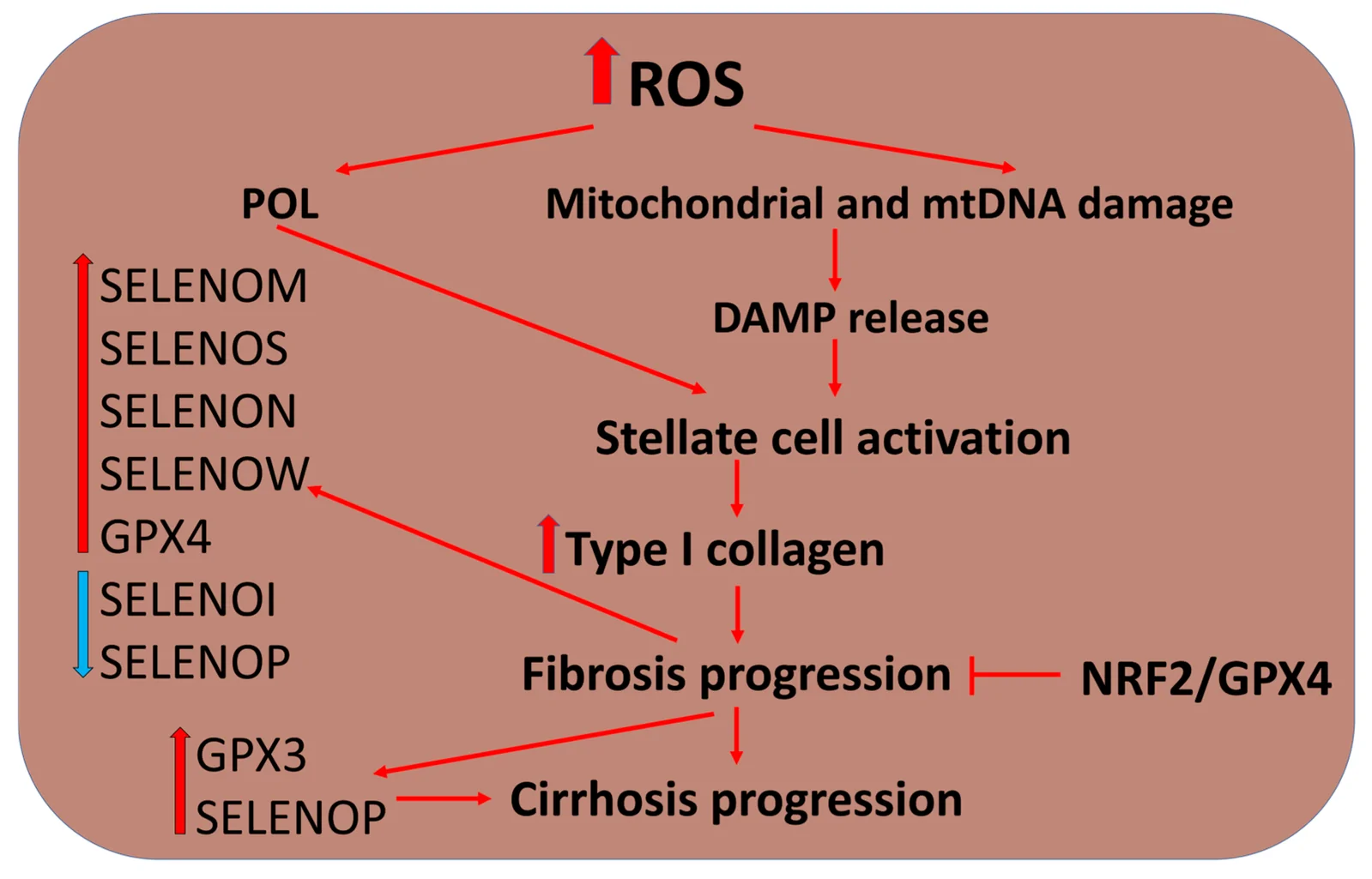
The main stages of fibrosis and cirrhosis progression caused by ROS generation.

The main stages of fibrosis and cirrhosis progression caused by ROS generation are shown schematically in Figure 5.

Increased levels of reactive oxygen species trigger two parallel processes: lipid peroxidation (POL) and mitochondrial/mtDNA damage. Mitochondrial damage leads to the release of deactivating molecular fragments (DAMPs). Both pathways activate hepatic stellate cells, which in turn activate type I collagen production. This leads to the progression of fibrosis and ultimately cirrhosis. During fibrosis, levels of the selenoproteins SELENOM, SELENOS, SELENON, SELENOW, and GPX4 increase, while levels of the selenoproteins SELENOI and SELENOP decrease. During the transition from fibrosis to cirrhosis, levels of GPX3 and SELENOP additionally increase. Activation of the NRF2/GPX4 pathway, which inhibits ferroptosis and inhibits the progression of fibrosis, should be considered a protective mechanism against fibrosis.

## 6. Hepatocellular Carcinoma (HCC)

HCC is the sixth most commonly diagnosed type of cancer and accounts for 75–85% of all liver cancer cases, arising from the malignant transformation of hepatocytes [231,232,233,234]. Risk factors for HCC include gender, race, chronic viral hepatitis, cirrhosis, inherited metabolic diseases, alcohol consumption, smoking, obesity, type 2 diabetes, and exposure to carcinogens such as aflatoxins [232,235]. However, oxidative stress also plays a significant role in the progression of HCC.

### 6.1. The Role of ROS in the Development of HCC

It is well known that at moderate concentrations, ROS activate the cancer cell survival signaling cascade (MAPK/ERK1/2, p38, c-Jun N-terminal kinase, etc.), while at high concentrations, ROS can induce cancer cell apoptosis [236,237,238,239,240,241]. Furthermore, ROS dynamically influence the tumor microenvironment, initiating angiogenesis, metastasis, and cancer cell survival at various concentrations. Oxidative stress promotes the development of HCC through both genetic and epigenetic changes, including alterations in the expression of oncogenes, tumor suppressors, and proinflammatory markers [242]. Thus, the main product of lipid peroxidation (4-hydroxy-trans-2-nonenal) forms DNA adducts and can contribute to mutations in the p53 tumor suppressor associated with HCC [243,244].

Furthermore, mitochondrial dysfunction plays an important role in the progression of HCC, since cancer cells use aerobic glycolysis rather than oxidative phosphorylation. Warburg suggested that this may be associated with mitochondrial defects [245,246,247]. This results in a disruption of electron flow in the electron transport chain, increasing ROS generation, which has negative consequences [248,249]. Excessive ROS accumulation also results in a decrease in mtDNA copy number, mtDNA mutations and deletions, and a decrease in mitochondrial transcription factor A (TFAM), which is necessary for maintaining mtDNA stability [250,251,252,253].

It is well known that ROS also act as a component of the relationship between chronic inflammation and HCC, affecting the levels of HIF-1α, NF-kB, inflammatory cytokines, growth factors, etc. [254,255,256,257]. In turn, inflammatory cells of the tumor microenvironment are also capable of generating excessive amounts of ROS through the regulation of the activity of redox enzymes: NADPH oxidase, xanthine oxidase, lipoxygenase, inducible nitric oxide synthase, etc. In addition, ROS contribute to the stimulation of metastasis by enhancing the epithelial–mesenchymal transition [258,259,260], and enhancing angiogenesis through the secretion of angiogenic modulators [261,262,263]. A recognized factor contributing to hepatocarcinogenesis is ER stress, which is closely associated with oxidative stress [264,265,266]. Furthermore, oxidative stress promotes the redistribution of cellular calcium from the ER to the cytosol, mitochondria, and nuclei, which contributes to increased mitochondrial membrane permeability, activation of calcium-dependent endonucleases, proteases, and lipases, and the release of pro-apoptotic factors, all of which contribute to hepatocyte death and can ultimately lead to HCC [267,268].

Thus, reactive free radicals and non-radical oxidants can attack and damage all cellular macromolecules, including proteins, nucleic acids, and lipids in hepatocytes. Protein oxidation can have negative functional consequences, while nucleic acid oxidation can lead to mutagenesis, which contributes to the progression of HCC.

**Figure 6:**
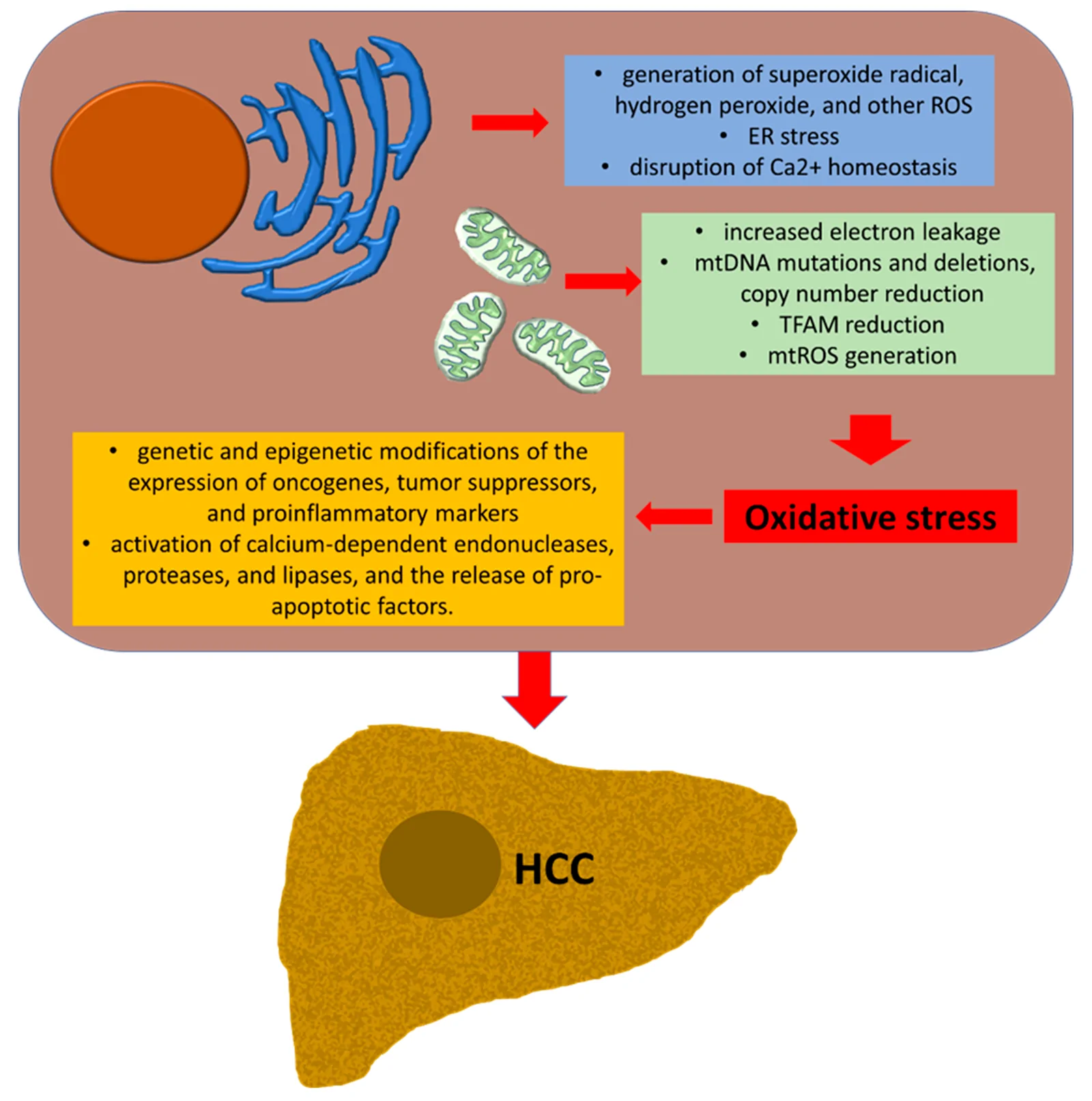
Molecular and cellular mechanisms of HCC pathogenesis induced by oxidative stress.

The main processes associated with the progression of oxidative stress that cause HCC are shown in Figure 6.

Excess ROS triggers mitochondrial dysfunction (Warburg effect), ER stress with disruption of calcium homeostasis, and macromolecular damage. These processes lead to hepatocyte death, genetic mutations (including p53), and the formation of an inflammatory tumor microenvironment, which collectively stimulates angiogenesis, epithelial–mesenchymal transition (EMT), metastasis, and HCC progression.

### 6.2. Antioxidant and Other Functions of Selenoproteins in HCC

Available data indicate that cancer progression correlates with a decrease in Se concentration in HCC tumor tissues, and that the antioxidant properties of selenoproteins play an important role in cancer protection. For example, gene expression profiling of HepG2 and Huh7 cells and normal hepatocytes using RT-qPCR revealed that in two HCC cell lines, decreased expression of three genes (DIO1, DIO2, and SELENOO) and increased expression of ten genes (GPX4, GPX7, SELENOK, SELENOM, SELENON, SELENOT, SELENOV, SELENOF, SELENOW, and TXNRD1) were observed [269].

GPXs, which are direct antioxidant enzymes, play an important role in maintaining cellular homeostasis by protecting cells from oxidative damage, which also extends to the field of oncology [270,271]. Some studies have shown that GPX2 is significantly elevated in HCC cells, where it is an important regulator of ROS, and inhibition of this enzyme can enhance the antitumor response of various drugs in the fight against HCC [272,273]. Thus, it was shown that downregulation of GPX2 expression significantly increased ROS levels in HepG2 cells, while overexpression of GPX2 significantly decreased ROS levels in Huh7 cells. Furthermore, this study showed that GPX2 plays an important role in tumor sensitivity to lenvatinib in HCC [274]. Another study demonstrated that GPX2 was overexpressed in the most metastatic rat HCC cell lines (N1 and L2 cells) [272]. When its activity was suppressed in HCC cells, significant inhibition of cell proliferation was observed after 24 and 48 h, accompanied by apoptosis but not cell cycle arrest. Reducing GPX2 activity also reduced the expression of mutated p53, migration and invasion, decreased matrix metalloprotease 9 secretion, and reduced the area of metastatic nodules in the lungs. This phenomenon likely describes a fundamental property of oncogenic reprogramming of tumor cells, in which GPX2 switches from its normal physiological function (protecting healthy tissue from damage) to promoting tumor survival and aggressiveness. Tumors intentionally overexpress GPX2 to maintain ROS levels at a precisely defined level: low enough to support survival, but high enough to stimulate growth signals.

Conversely, another member of the glutathione peroxidase family, GPX3, is characterized by decreased expression in HCC cells and tumor tissues, which significantly correlates with poor overall survival in HCC patients. Among the HCC cell lines, GPX3 expression was significantly lower in the metastatic HCC cell line (MHCC97L) compared with the non-metastatic HCC cell lines (Hep3B and Huh7) [274]. This study also showed that GPX3 overexpression suppressed the invasiveness of HCC cells by inhibiting EMT through the Erk-NF-kB-SIP1 signaling pathway. Another study showed that the antitumor function of GPX3 could be mediated through the JNK-cJun-MMP2 signaling pathway. Furthermore, the authors also proved that decreased GPX3 levels in small liver grafts were significantly associated with HCC invasiveness in a rat model, and lower plasma GPX3 levels were an independent predictor of poor overall survival in HCC patients after liver transplantation [275].

GPX4 overexpression predicts poor prognosis in patients with HCC by protecting cells from oxidative stress and reducing intracellular ROS levels, thereby promoting tumor cell proliferation, migration, angiogenesis, and immune cell infiltration [276,277,278].

The thioredoxin system is also activated in cancer, and TXNRD1 is overexpressed in various solid tumors, such as HCC. Therefore, TXNRD1 has been proposed as a prognostic marker for patients with human HCC [279,280]. Increased TXNRD1 expression in clinical samples was positively correlated with advanced clinical stage and poorer patient survival. One study demonstrated that TXNRD1 expression is regulated through the KEAP1-NRF2 pathway [280]. NRF2 is known to be a key regulator of oxidative stress, the expression of which is suppressed by KEAP1 (kelch-like ECH-associated protein 1) in normal cells [281,282]. However, ROS can disrupt the interaction between KEAP1 and NRF2, which promotes NRF2 translocation to the nucleus to activate antioxidant defense genes. In NRF2-knockdown HCC cell lines, a decrease in TXNRD1 levels was observed, which proved that NRF2 can be considered a transcription factor of TXNRD1 in HCC [280]. Knockdown of TXNRD1 resulted in significant suppression of cell proliferation, cell cycle arrest, and suppression of tumor formation, which was caused by the action of accumulated ROS. Furthermore, it was found that TXNRD1-deficient tumors had significantly increased expression of GPX2 and enzymes involved in glutathione metabolism (GSH, GST-α1, GSR, and GCLC) [283]. Thus, the authors suggested that TXNRD1-deficient livers provide additional antioxidant support.

Using in situ hybridization and immunohistochemistry, it was found that SELENOP mRNA expression was significantly lower in HCC tissues than in healthy tissues [284,285]. This contributed to the inhibition of proliferation of these cancer cells, inhibition of ROS, and increased expression of GPX1 in these cells [286]. When studying the molecular mechanisms of SELENOP expression suppression in HCC cells, it was found that the transcription factor NRF2 was involved in this process, which promotes increased expression of the canonical targets NQO1 and TXNRD1, while suppressing SELENOP expression. Furthermore, overexpression of recombinant SELENOP in HepG2 cells revealed a lower basal SELENOP expression level than in HepG2 cells; deficiency of this selenoprotein was accompanied by accumulation of lipid hydroperoxides and increased resistance to ferroptosis [269].

Expression of selenium-binding protein 1 (SELENBP1) in tissue samples from HCC patients. SELENBP1 is reduced in liver tissue from HCC patients [287]. When assessing liver selenium levels in HCC patient tissue samples stratified by grade using atomic absorption spectrometry, mean liver selenium concentrations were lower in patients with the highest grade of malignancy in the following order: grade I > grade II > grade III. Moreover, these values correlated with SELENBP1-related immunohistochemical indices [288]. Thus, assessment of Se and SELENBP1 concentrations is particularly useful for improving prognosis in prospective liver biopsy in patients with cirrhosis and HCC.

Immunohistochemistry showed that high expression of SELENOM was observed in the cytoplasm of hepatocytes in HCC, and that as HCC progressed (from stage 1 to 3), the SELENOM level increased [289]. When comparing the expression levels of this selenoprotein in two HCC cell lines, HepG2 and Huh7, it was shown that it was expressed to a greater extent in the HepG2 hepatoma cell line [111,290]. Previously, we also showed that in HepG2 cells, 24 and 48 h exposure to selenium nanoparticles (SeNPs) contributed to a significant suppression of SELENOM mRNA expression, which correlated with a decrease in the viability of these cells [236]. In addition to SELENOM, we also examined the expression patterns of other ER-resident selenoproteins, finding that a similar trend toward decreased mRNA expression was characteristic of two other selenoproteins: SELENOS and SELENOF.

In another study, using two HCC cell lines, HepG2 and Huh7, a correlation was found between selenium availability and the cellular abundance of two proteins, GPX1 and SELENOK [291]. Furthermore, after 24 h stimulation with various selenite concentrations (0.25, 0.5, and 1 μM), a significant increase in the expression of these selenoproteins was observed even at low doses of sodium selenite. This correlated with a decrease in the proinflammatory markers IL-6, IL-8, and IL-17, indicating inhibition of tumor invasiveness.

**Figure 7:**
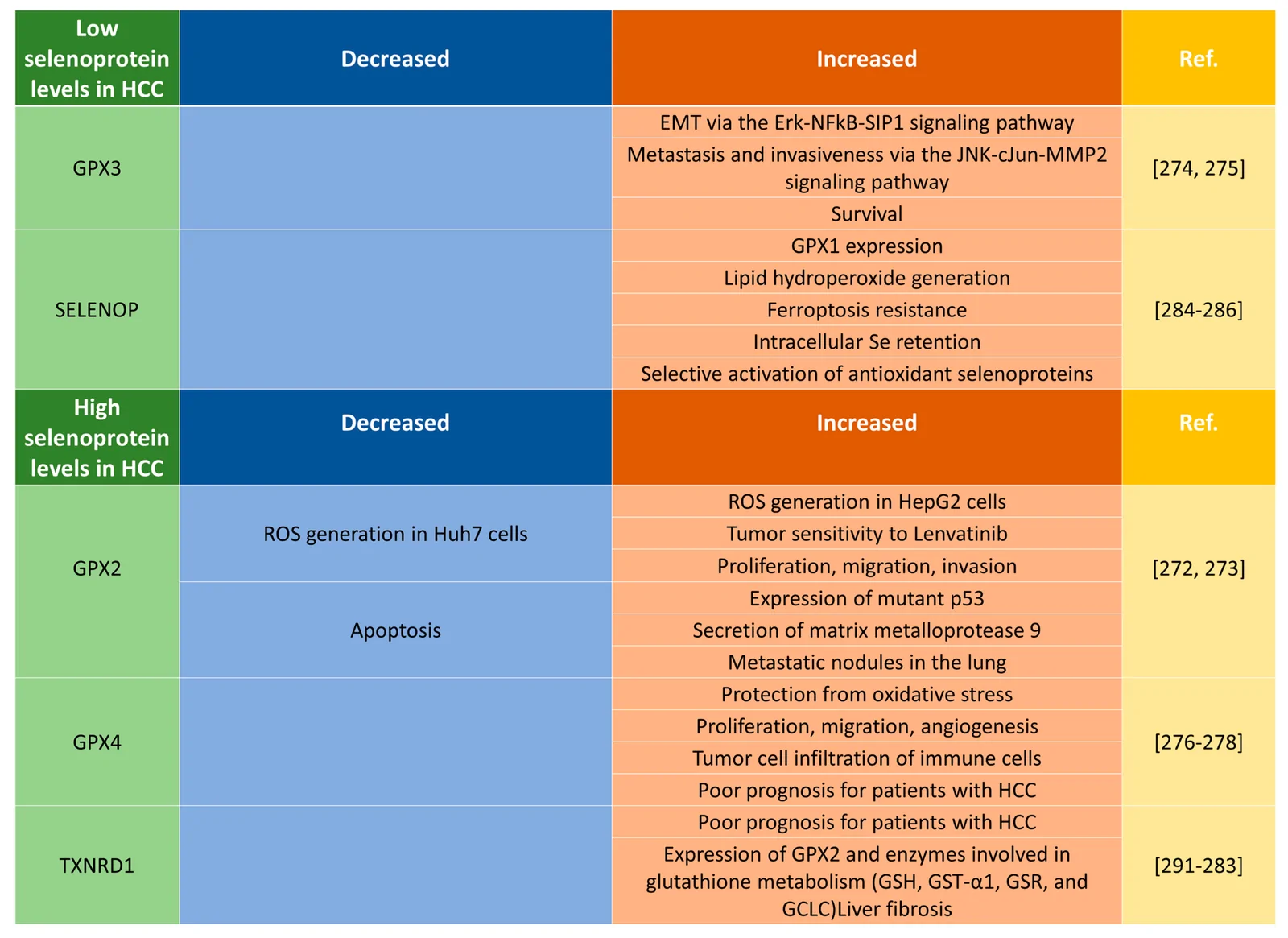
Pathological processes in the liver caused by changes in selenoprotein levels, leading to the development of HCC.

The key functions of selenoproteins in the regulation of processes associated with HCC are presented in Figure 7.

Selenoproteins whose expression in the liver is increased or decreased are separated. Consequences that depend on the level of selenoprotein expression are color-coded: if certain processes are enhanced (orange), if certain processes are suppressed or inhibited (blue). EMT—epithelial-mesenchymal transition; Erk—extracellular signal-regulated kinase; NF-κB—nuclear factor kappa B; SIP1—Smad-interacting protein 1; GSH—reduced glutathione; GCLC—glutamate-cysteine ligase catalytic subunit; GST—α1-glutathione S-transferase alpha 1; GSR—glutathione-disulfide reductase.

## 7. Prospects and Limitations of the Use of Selenoproteins in the Treatment of Various Liver Pathologies

The liver is constantly exposed to high oxidative stress, as it plays a crucial role in the safe removal of endogenous and exogenous toxins. Insufficient Se intake and low expression of the corresponding selenoproteins involved in antioxidant defense may contribute to an increased risk of damage and malignant transformation of hepatocytes and, consequently, a higher incidence of various liver pathologies. Selenoproteins can neutralize ROS, preventing damage to hepatocyte membranes, help restore mitochondrial homeostasis, reduce steatosis, and suppress the activity of collagen-producing cells, inhibiting the development of fibrosis. Furthermore, maintaining optimal selenoprotein levels can reduce tumor cell proliferation and stimulate apoptosis. However, there is a narrow range between the therapeutic level of Se and its toxicity (selenosis). Conversely, selenium overdose can cause oxidative stress and liver damage, triggering metabolic disorders, including insulin resistance. Furthermore, it is well known that during severe systemic inflammatory reactions (the acute phase), the liver reduces SELENOP synthesis, which is also a protective mechanism aimed at redistributing the micronutrient. Another limitation of the use of selenoproteins in the treatment of liver pathologies is the limited clinical evidence base, as many of their effects have been studied primarily in vitro and in animal models. Large-scale clinical trials sometimes yield conflicting results, which is why selenium is still not included in strict treatment protocols for MAFLD. A personalized approach is necessary to realize the therapeutic potential, as the baseline selenium status in patients with cirrhosis or hepatitis can vary greatly.

**Figure 8:**
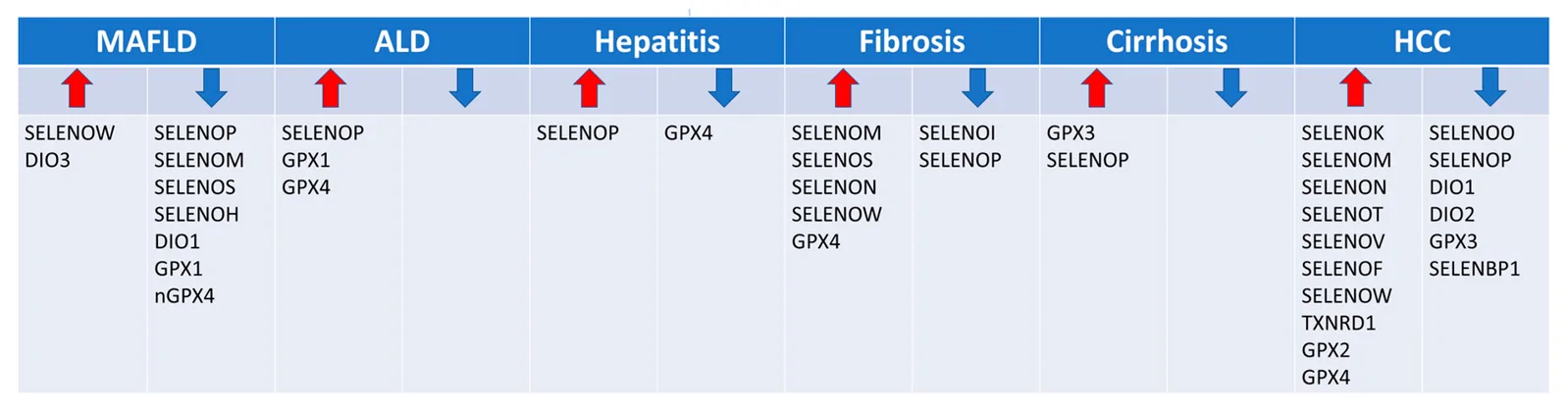
Schematic representation of selenoprotein levels in various pathologies. High selenoprotein levels are indicated by the red arrow, low levels by the blue arrow.

Summarizing the data described in the review regarding the levels of selenoproteins in various pathologies, the following diagram was drawn up and is presented in Figure 8.

The presented data suggest that the expression of a number of selenoproteins decreases or increases depending on the disease, and this does not always correlate with disease progression. For example, SELENOP levels are decreased in MAFLD, increased in hepatitis, decreased in fibrosis, increased in cirrhosis, and decreased in HCC. Firstly, a key limitation of this review due to the insufficient amount of data is that protein levels are presented at different levels: transcriptional, translational, serum, tissue, and cellular. For example, SELENOP levels in MAFLD, ALD, and cirrhosis are presented as protein content in the blood of animals, whereas in hepatitis, fibrosis, and HCC, data on SELENOP mRNA expression are presented. It can be hypothesized that decreased SELENOP in serum in fatty degeneration may be caused by disruption of the ER and Golgi apparatus structure due to the fact that hepatocytes contain a lot of fat. This may disrupt the normal secretion of SELENOP into the blood. Increased mRNA expression in hepatitis is likely associated with the influx of inflammatory cytokines (IL-6, TNF-alpha). These cytokines activate STAT3, which directly stimulates the SELENOP gene promoter [292]. Increased serum protein levels in cirrhosis are possible. They are associated with metabolic disorders and severe insulin resistance, which promotes the synthesis of SELENOP, which can be suppressed by insulin [293,294]. In the case of SELENOM, data on changes in expression levels in the liver are presented at the transcriptional and translational levels; however, in MAFLD, these levels are reduced, while in Fibrosis and HCC, on the contrary, they are increased. It is possible that, in this case, increased SELENOM levels correlate with the severity of liver pathologies. A decrease in SELENOM at the transcriptional and translational levels may be a primary defect that makes hepatocytes defenseless against lipotoxicity, triggering their death. Activation of stellate cells during fibrosis requires a restructuring of calcium signaling, for which SELENOM is responsible, in particular [295]. Furthermore, it is well known that SELENOM levels are high in many cancers, including HCC, which is primarily associated with the activation of MAPK pathways and the regulation of oxidative stress and ER stress [296,297,298]. Interestingly, SELENOW expression levels in the liver are high in MAFLD, Fibrosis, and HCC. Since SELENOW is highly sensitive to changes in the ratio of oxidized to reduced glutathione [111] it is activated immediately and remains consistently high throughout all stages as a universal cytoplasmic antioxidant, attempting to prevent oxidative DNA damage. Levels of glutathione peroxidases and thioredoxin reductases also do not correlate with the severity of liver pathologies, according to available data. Since the pattern of various selenium-containing GPXs and TXNRDs varies in different pathologies, it is possible that there is an effect of interchangeability between enzymes belonging to the same class.

Thus, the currently available scientific data regarding the levels of various selenoproteins in various liver pathologies do not allow us to confidently identify patterns and correlations between the expression of these proteins depending on the severity of liver disease. However, the various functions of selenoproteins presented in this review provide grounds for considering the important role of these proteins in regulating processes associated with liver disease.

## Data Availability

No new data were created or analyzed in this study. Data sharing is not applicable to this article.

## Abbreviations

ACOX1	Acyl-CoA oxidase 1
ACSL4	Acyl-CoA Synthetase Long-Chain Family Member 4
ALD	Alcoholic liver disease
ALOX15	Arachidonate 15-lipoxygenase
ALT	Alanine Aminotransferase
AMPK	Adenosine Monophosphate-activated Kinase
AST	Aspartate Transaminase
CAT	Catalase
cGAS	cyclic GMP-AMP synthase
CYP450	Cytochrome P450
DIO	deiodinase
DUSP	Dual Specificity Phosphatase
ER	Endoplasmic Reticulum
ERK	Extracellular signal-Regulated Kinase ½
FGF21	Fibroblast Growth Factor 21
FOXO3	Forkhead box O3
GPX	Glutathione Peroxidase
HIF-1α	Hypoxia-Inducible Factor 1-alpha
iNOS	Inducible Nitric Oxide Synthase
MAFLD	Metabolic Fatty Liver Disease
MAPK	Mitogen-Activated Protein Kinase
mDAMPs	Mitochondrial Damage-Associated Molecular Patterns
MFN2	Mitochondrial Fusion Protein
mtDNA	mitochondrial DNA
NADPH	Nicotinamide Adenine Dinucleotide Phosphate
MAFLD	Non-Alcoholic Fatty Liver Disease
NASH	Non-Alcoholic Steatohepatitis
NO	NADPH Oxidases
NRF2	Nuclear Factor Erythroid 2-Related Factor 2
NS3	Nonstructural protein 3
RNS	Reactive Nitrogen Species
SELENO	Selenoprotein
SOD	Superoxide Dismutase
STAT3	Signal Transducer and Activator of Transcription 3
STING	Stimulator of Interferon Genes
TFR1	Transferrin Receptor 1
TNF-α	Tumor Necrosis Factor-alpha
Trx	Thioredoxin
TXNR	Thioredoxin Reductase
4-HNE	4-hydroxy-2-nonenal
